# Four‐year field study reveals variable effects of phytohormone‐ and natural‐based elicitors on anthocyanin metabolism in Tempranillo grapes

**DOI:** 10.1002/jsfa.70050

**Published:** 2025-08-08

**Authors:** Sara Crespo‐Martínez, Nazareth Torres, Maite Loidi, Carlos Miranda, Jesús Astráin, L. Gonzaga Santesteban, Jorge Urrestarazu

**Affiliations:** ^1^ Deparment of Agronomy, Biotechnology and Food Science Public University of Navarre, Campus Arrosadia Pamplona Spain; ^2^ Institute for Multidisciplinary Research in Applied Biology (IMAB), Jerónimo de Ayanz, Campus Arrosadia Pamplona Spain; ^3^ Bodegas Pagos de Araiz Olite Spain

**Keywords:** anthocyanins, grapevine, RT‐PCR, natural‐based elicitors, phytohormone‐based elicitors

## Abstract

**OBJECTIVES/BACKGROUND:**

Climate change has raised concerns about the imbalance between anthocyanin and sugar levels in grapevines. High temperatures tend to reduce anthocyanin content by inhibiting its synthesis and increasing degradation, while simultaneously enhancing sugar accumulation. The application of elicitors, which promote the anthocyanin biosynthetic pathway, may help alleviate this imbalance.

**METHODS:**

To investigate this, a four‐year field trial was conducted on *Vitis vinifera* cv. Tempranillo to assess the effects of four commercial products: two natural‐based (an antioxidant‐mannitol and an alginic acid‐mannitol products) and two phytohormone‐based (an abscisic acid and an ethephon‐based products). The study evaluated the effect of these treatments on the phenyl propanoid pathway gene expression, various yield parameters, and the content of primary and secondary metabolites.

**RESULTS:**

Although none of the treatments consistently increased anthocyanin levels across seasons, the results varied depending on the year. Two products influenced some parameters in specific seasons: the ethephon‐based product, which influenced the anthocyanin‐to‐sugar ratio through the reduction of TSS and induced the expression of key anthocyanin genes, and the natural‐based antioxidant‐mannitol product, which transiently increased the expression of some phenylpropanoid genes.

**CONCLUSIONS:**

None of the treatments increased anthocyanin levels across seasons. Ethephon (Fruitel) showed greater effectiveness under temperature stress and reduced sugar accumulation, which may be advantageous in a warming climate. Natural elicitors like antioxidant–mannitol elicitor (Vitalfit) had short‐term effects on gene expression but no lasting impact on anthocyanins. Further research is needed to assess their influence on other polyphenols and their potential for commercial use. © 2025 The Author(s). *Journal of the Science of Food and Agriculture* published by John Wiley & Sons Ltd on behalf of Society of Chemical Industry.

## INTRODUCTION

Phenolic compounds are crucial components of berries (*Vitis vinifera* L.) due to their significant impact on wine quality.^1^ These compounds are categorized into flavonoids (mainly anthocyanins, flavonols, and flavan‐3‐ol monomers) and nonflavonoids (hydroxycinnamic acids, hydroxybenzoic acids, volatile phenols, and stilbenes). Among the flavonoids, anthocyanins and flavonols play a major role in determining the colour, stability, and hue of wine through co‐pigmentation processes.^2^ Additionally, proanthocyanidins (also known as condensed tannins) which are polymerized forms of flavan‐3‐ols found in the seeds and skins of berries, contribute to the wine's colour and mouthfeel.^3^, ^4^

Climate change has raised significant concerns regarding the reduction in berry colour over recent decades. Research indicates that high temperatures can lead to a significant reduction in total anthocyanin concentration in grape berries.^5^, ^6^ Additionally, the relationship between anthocyanins and total soluble solids (TSS) can be decoupled at elevated temperatures, leading to a delayed onset of anthocyanin accumulation.^7^ This phenomenon is particularly evident in the widely planted Spanish cultivar Tempranillo, which is notably sensitive to these climatic changes.^8^, ^9^, ^10^ Moreover, the decoupling between sugar and anthocyanin accumulation now represents one of the key viticultural challenges, particularly in warmer regions.

Different strategies may be adopted to deal with the polyphenol deficit in berries grown in warm areas. For new plantations, selecting clones and rootstocks that provide adequate phenolic profiles under such conditions is critical.^11^ In established vineyards, various cultural practices can be implemented, including canopy management,^12^, ^13^, ^14^ cluster thinning,^14^, ^15^, ^16^ and deficit irrigation^17^, ^18^, ^19^, ^20^ and furthermore, there is an increasing interest for exploring new products that upregulate phenolic metabolism to prevent the low anthocyanin content in berries at harvest.^21^ A wide variety of molecules known as elicitors can serve as phenol enhancers. Those molecules can be endogenous, being components of the plant signalling system, or exogenous, which are mainly related to plant pathogen interactions.^22^

Plant hormones (PH) are endogenous elicitors that play a pivotal role in the synthesis of phenolic and aromatic compounds in grapevines. Key hormones as jasmonic acid (JA), salicylic acid (SA), and ethylene (ET), are essential to plant defence mechanisms against biotic stresses.^23^, ^24^ These hormones initiate a cascade of signalling pathways that ultimately enhance the expression of genes involved in the polyphenol biosynthesis, including *VvPAL*, *VvMYB*, *VvCHS*, *VvCHI*, *VvDFR*, *VvF3H*, *VvUFGT*, and *VvANS*.^25^, ^26^, ^27^, ^28^, ^29^, ^30^, ^31^ Phenolic compounds, in turn, act as antioxidants, playing a critical role in plant defence.^32^ Among these, methyl jasmonate (MeJ), a derivative of JA, and benzothiadiazole (BTH), an SA analogue, have been shown to enhance anthocyanin accumulation in grapevines.^21^, ^33^, ^34^, ^35^, ^36^, ^37^, ^38^, ^39^, ^40^, ^41^, ^42^, ^43^, ^44^ However, the widespread use of these elicitors is often limited by economic constraints and ecologic‐logistical challenges.^39^, ^45^ In this regard, abscisic acid (ABA)^46^, ^47^, ^48^, ^49^ and ET^50^, ^51^, ^52^, ^53^, ^54^ emerged as cost‐effective alternatives with significant potential to boost anthocyanin production. ABA applications have outperformed MeJ and BTH in anthocyanin accumulation in grape skins in some studies.^21^, ^28^ Despite their benefits, ABA and ET generally accelerate ripening, which can lead to increased sugar content—an undesirable outcome in the context of global warming.^55^ Furthermore, PH treatments are not without drawbacks. Issues such as hormonal crosstalk,^56^, ^57^ challenges in treatment timing and adjustment,^35^, ^50^, ^58^, ^59^, ^60^, ^61^, ^62^ and its prohibition in organic crops, limit their practical application.

Exogenous elicitors are molecules that are not naturally synthesized by the organism itself. These molecules bind to receptors in plant cell membranes, triggering a defensive signalling cascade upon detection. This cascade is associated with stress responses and ultimately leads to the synthesis of PH.^24^, ^63^ Its relevance for crop elicitation stems from its operational simplicity, cost‐effectiveness, and improved safety for both environment and human health.^21^, ^64^, ^65^, ^66^ Exogenous elicitors can be classified based on their nature into two categories: abiotic (e.g., ions) and biotic (e.g., fungal or plant components). Among abiotic elicitors, such as heavy metals, ions can either act as cofactors or activate plant defence mechanisms. Biotic elicitors are generally preferred for eliciting anthocyanin production in grapevines.^67^, ^68^ Fungal derived elicitors stimulate plant secondary metabolism through a signalling cascade that triggers the synthesis of ET or SA.^69^ Among them, yeast extract elicitation has shown positive results in Sangiovese and Tempranillo vines^40^, ^70^ and chitosan increased polyphenols in Tinto Cão berries but showed no impact on Tempranillo.^40^, ^71^ Among plant‐based elicitors, several have demonstrated potential in grapevines through different mechanisms: (i) pectin‐derived oligosaccharides (PDOs), which trigger plant defence responses and stimulate the synthesis of SA and JA,^72^ have been shown to enhance anthocyanin metabolism in Cabernet Sauvignon and Flame Seedless,^73^, ^74^ and (ii) mannitol or alginic acid foliar application, which alleviates osmotic stress and promotes the synthesis of ABA or SA,^75^ enriched the phenol profile in Touriga Nacional grapes^76^ and Marselan grapes^77^ respectively. However, the effects of these alternatives can be inconsistent, often influenced by seasonal conditions, grapevine varieties, or other environmental factors. Moreover, some treatments may negatively impact other grape components, necessitating further research to refine their application.^45^

Furthermore, given that elicitors may preferentially activate certain branches of the polyphenol pathway (e.g., stilbene *vs* anthocyanin synthesis), understanding their specificity is crucial in interpreting interannual variability.

In the scope of the abovementioned research, this study aimed to investigate the effects of foliar applications of four different elicitors—two PH‐based and two natural‐based—on the expression of phenolic pathway genes and the resulting berry quality over four growing seasons.

## MATERIAL AND METHODS

### Plant material and experimental design

A four‐season field trial was conducted in a commercial vineyard located in Olite, Navarre, Spain (42°25′20.9″N 1°40′48.8″W), owned by Pagos de Araiz winery. The vineyard was established in 2001 with *Vitis vinifera* cv. Tempranillo (clone RJ43) grafted onto 140 Ruggeri rootstocks. Vines were spaced 2.50 × 1.10 m (row × vine) and trained to a two‐sided Royat cordon system, with rows‐oriented East–West. Mid‐veraison dates varied across seasons: July 27 (2020), August 4 (2021), July 14 (2022), and July 22 (2023). Technological ripeness was reached on September 1 (2020), September 7 (2021), August 30 (2022), and August 31 (2023).

The trial tested four elicitors: two PH‐based (Protone and Fruitel) and two natural‐based products (Vitalfit and SM6). The PH‐based products, one containing ABA and the other ET, were selected due to their direct involvement in the ripening process, as well as their affordability for grape farms. A natural product elicitor, which could substitute PHs, was included in order to approach a more sustainable plant protection for organic agriculture. Vitalfit was chosen for its composition as an antioxidant promoter, which is known to stimulate anthocyanin metabolism, and its popularity among farmers in the region. Antioxidant‐mannitol elicitor (Vitalfit) was included in all four seasons, while SM6, which contains a higher mannitol concentration, was included in the 2022 and 2023 trials to assess whether increasing mannitol levels would enhance its effects. Elicitors were compared against untreated vines (control). Product compositions were (i) 10.4% S‐abscisic acid (Protone, Valent Bioscience CO), (ii) 48% 2‐chloroethephonylphosphonic acid (ethephon) (Fruitel, Bayer), (iii) Vitalfit complex +3% NH_2_ + 15% P + 2.8% Mn + 1% Zn + 0.2% mannitol (Vitalfit, Timac Agro) and (iv) 6% alginic acid +2% mannitol from *Ascophyllum nodosum* (SM6, Plymag). Applications were conducted according to the manufacturers' recommendations. The natural‐based products were applied three times: 1 week before veraison (BBCH73), at mid‐veraison (BBCH83), and 3 weeks post‐veraison (BBCH85).^78^ The PH‐based products required a single application at mid‐veraison (BBCH83). Applications were performed with an atomizer using doses of 2.5 L/ha for Vitalfit, 1–3 L/ha for SM6, 4 L/ha for Protone, and 1 L/ha for Fruitel (Table 1). The experimental design comprised four randomized blocks, each containing 30 vines, of which the 10 central vines were used for destructive sampling.

**Table 1:** Treatment Ethephon elicitor (Fruitel), ABA elicitor (Protone), antioxidant‐mannitol elicitor (Vitalfit), and alginic acid‐mannitol elicitor (SM6) and sampling timeline

Treatment	Company	Elicitor components	Treatment application times			
			Pea‐sized berries BBCH75	Berries develop colour BBCH83	Softening of berries BBCH85	Berries ripe for harvest BBCH89
Control	‐	‐				
Vitalfit	Timac Agro	VITALFIT complex, urea‐N (3%), P (15%), Mn (2.8%), Zn (1%), and mannitol (0.2%)				
SM6	Plymag	Alginic acid (6%) and mannitol (2%)				
Fruitel	Valent Biosciences	S‐abscisic acid (10.4%)				
Protone	Bayer	Ethephon (48%)				
			Sampling 1 Veraison Gene expression		Sampling 2 Ripening Gene expression	Sampling 3 Harvest Grape composition

Weather data were obtained from the SIAR weather station in Olite (#11, Navarra, Spain) located close to the research site.

### RNA extraction, cDNA synthesis and qRT‐PCR

For gene expression analysis, samples of 25–30 berries per treatment were collected during ripening, immediately frozen in liquid nitrogen, and stored at −80 °C until extraction. Samples were homogenized under frozen conditions with a mortar and subsequently processed with a microdismembrator (B. Braun, Germany) at 1900 rpm for 1 min. RNA was extracted from 400 mg of tissue using a modified CTAB method protocol. CTAB buffer was enriched with 2% PVPP‐40 and 5% β‐mercaptoethanol. Samples were vortexed in 1 mL lysis buffer, incubated at 65 °C (700 rpm, 20 min), and purified three times with chloroform alcohol (24:1 v/v).^79^, ^80^ After precipitation with sodium acetate (3 M, pH 5.2) and isopropanol, RNA pellets were washed with 70% ethanol and purified using the Plant Total RNA Kit (Sigma‐Aldrich, USA). RNA quality and concentration were assessed with a FLUOstar Omega Microplate Reader (BMG Labtech, Germany).

A total of 500 ng of extracted RNA were treated with the PrimeScriptTM RT Reagment Kit (Perfect Real Time) (Takara, Japan) and reverse‐transcribed using a T100 Thermal Cycler (Bio‐Rad Laboratories, USA) according to the manufacturer's instructions.

cDNA was synthesized using the PrimeScript™ RT Reagent Kit (Perfect Real Time) (Takara, Japan) from 500 ng of RNA, with reactions performed on a T100 Thermal Cycler (Bio‐Rad, USA). Quantitative PCR was carried out with 10 ng cDNA, TB Green Premix Ex Taq II (Takara, Japan), and primers (Thermo Fisher, USA) at 0.4 μM concentration. PCR was run on a StepOnePlus System (Applied Biosystems, USA), with each sample analysed in biological triplicates and technical triplicates. Actin was used as the reference gene. Key genes involved in the phenylpropanoid (PP) pathway were assessed, including *VvPAL*, *VvDFR*, *VvLDOX*, *VvUFGT*, *VvAOMT*, and *VvANR* (Table 2). Relative expression was calculated using the 2^−ΔΔCt^ method being primer efficiency between 90% and 110%.^81^

**Table 2:** List of the primers and melting temperatures used for real time RT‐PCR

Gene	Encoded protein	Primer pair (F, R)	References	*T*_m_ (°C)
*VvPAL*	Phenylalanine ammonia lyase	TGCTGACTGGTGAAAAGGTG CGTTCCAAGCACTGAGACAA	(Belhadj *et al*.)^33^	60
*VvDFR*	Dihydroflavonol reductase	GAAACCTGTAGATGGCAGGA GGCCAAATCAAACTACCAGA	(Jeong *et al*., 2004)^82^	56
*VvLDOX*	Leucoanthocyanidin dioxigenase	ACTCTTTGGGGATTGACTGG AGGGAAGGGAAAACAAGTAG	(Jeong *et al*., 2004)^82^	56
*VvUFGT*	UDP‐glucose:flavonoid 3‐O‐glucosyl transferase	AATCTGAGAGCCCTAAGAGA GGTGGTACAAGCAACAGTTC	(Goto‐Yamamoto *et al*., 2002)^83^	60
*VvAOMT*	Anthocyanin O‐methyl transferase	CTCTGCAGGCGCCTCTATTA CCCAAAACAGAGTCTGGACA	(Hugueney *et al*., 2009)^84^	58
*VvANR*	Anthocyanidin reductase	AGCAGGTTGCGACTTTGTCT ACCAGACCTGTCCCATCAAG	(Castellarin *et al*., 2007)^85^	60
*VvACTIN*	Actin	TGTGCTTAGTGGTGGGTCAA ATCTGCTGGAAGGTGCTGAG	(Griesser *et al*., 2018)^86^	60

### Yield parameters

Yield parameters number of clusters per vine, cluster weight and yield (kg/plant) were measured in the seasons 2021 and 2023 and berry weight in the four seasons (2020–2023). When harvest, number of clusters per vine were counted and weighted. Cluster weight was obtained as yield/number of clusters. Berry weight was obtained from 100 berries were randomly collected from the central 10 vines of each replicate (*n* = 6) across both growing seasons. The fresh samples were weighed to determine the average berry weight.

### Primary metabolites

A total of 100 berry samples were homogenized using a Classic 8‐Speed Blender (Oster, USA) at full speed. A portion of this homogenate (100 g) was macerated for 1 h at room temperature (22 °C) and subsequently centrifuged at 10000 rpm using a Hettich MIKRO 200/200 R centrifuge (Hettich, Germany) at 4 °C. The resulting supernatant was used to analyse the grape composition.

To measure total soluble solids (TSS) expressed as °Brix, a high‐precision, temperature‐compensating refractometer RFM840 (Bellingham + Stanley, UK) was used. The pH was assessed using a calibrated pH meter (pH‐Buretten 24, Crison Instruments, Spain) in combination with an electrode with temperature sensor (50 21 T, Crison Instruments, Spain). Titratable acidity (TA) was determined through titration (OIV, n.d.‐a), L‐Malic acid was analysed by enzymatic methods (OIV, n.d.‐c), tartaric acid was analysed by gravimetric methods (OIV, n.d.‐b) and Potassium was analysed by absorption spectrophotometry (OIV, n.d.‐d). All these analyses were performed according to the OIV standards. Free amino nitrogen (FAN) was determined enzymatically with the Sigma ammonia assay kit following manufacturer instructions (Sigma‐Aldrich, USA) using a spectrophotometer Cary 50 (Varian Medical Systems, Inc., USA).

### Phenolic compounds

Phenolic compounds were quantified using the Cromoenos™ method (Bioenos, Spain), a rapid and accurate technique for determining phenolic content and colour.^87^ At ripening, 200 berries per replicate (*n* = 6) were collected. Following the method, samples were blended (Oster, USA), and 40 mL aliquots were heated in a thermoextractor to 80 °C (~2 min). The samples were centrifuged (13 400 rpm, 2 min) with a centrifuge Mikro 200/200R, (Hettich), and 60 μL of the supernatant was diluted with 2% (v/v) HCl to 4 mL. Absorbance at 520 and 280 nm was measured with a spectrophotometer BioMate 3 (Thermo Fisher Scientific, USA). Using the software provided by Bioenos, total anthocyanins, extractable anthocyanins, tannins, total polyphenol index (TPI), and phenolic maturity index (PMI) were calculated based on absorbances and grape composition data (e.g., pH, TA, cultivar).

Phenolic compounds of grapes harvested in the season 2022 were further analysed by Ultra‐high‐performance liquid chromatography‐MS/MS (UHPLC–MS/MS) at ripening. The analysis were carried out at the Analysis Service of the Instituto de Ciencias de la Vid y del Vino (ICVV) following the procedure of Royo^88^ with the improvements of Fernández‐Zurbano.^89^

### Statistical analysis

Statistical analyses were conducted with IBM SPSS Statistics 27.^90^ Gene relative expressions data outliers were removed using the Grubbs method.^91^ Then, data was analysed by using the one‐way analysis of variance (ANOVA) and Tukey's post‐hoc test (*P* ≤ 0.05), after assessing the normality of the data. Heatmaps were constructed with the tool Heatmapper.^92^ Data in supplementary material is shown as means ± standard errors (SE). Yield parameters, primary metabolites, and phenolic compounds contents were analysed by using the one‐way analysis of variance (ANOVA) and Tukey's post‐hoc test (*P* ≤ 0.05), after assessing the normality of the data. As for the ratio anthocyanin content/TSS, yearly data are standardized as $\frac{\frac{\left(\frac{\mathrm{mg}\;\text{anthocyanin}}{l}\right)\text{treatment}}{\left(\frac{\mathrm{mg}\;\text{anthocyanin}}{l}\right)\text{control}}}{\frac{\left({}^{\circ}\text{brix}\right)\text{treatment}}{\left({}^{\circ}\text{brix}\right)\text{control}}}$.

## RESULTS

### Gene expression of the main genes in the polyphenol pathway

#### Veraison

**Figure 1:**
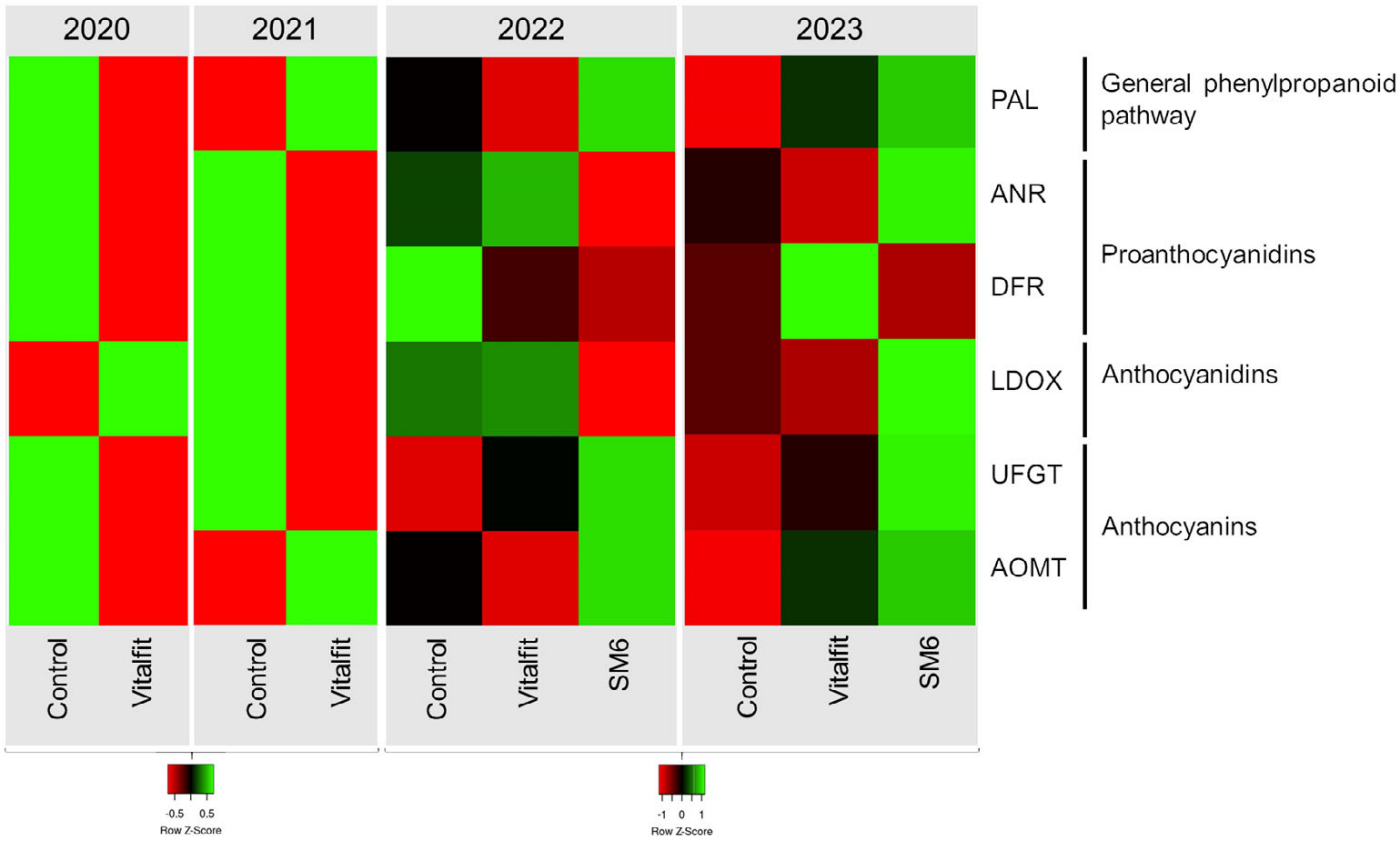
Heatmap of the expression analysis of the genes *VvPAL*, *VvDFR*, *VvANR*, *VvLDOX*, *VvUFGT* and *VvAOMT* at veraison (>60% of berries coloured) in the seasons 2020–2024. The treatments analysed were control, the antioxidant‐mannitol elicitor (Vitalfit), and the alginic acid‐mannitol (SM6).

At veraison, were analysed only the control and natural‐based elicitor treated berries as the PH‐based products were applied later, after veraison (Fig. 1 and Supporting Information, Fig. S1). As mentioned, the antioxidant‐mannitol elicitor (Vitalfit) was applied over 4 years, whereas the alginic acid‐mannitol elicitor (SM6) was applied in 2022 and 2023.

Overall, no significant differences were obtained after the first application of the natural‐based elicitors into the polyphenol gene activity. The only significant difference was reached in 2022 season, when the gene *VvPAL*, part of the general PP pathway, showed significantly lower expression in the antioxidant‐mannitol (Vitalfit) treated berries. In 2020, 2021 and 2022, although no statistical differences were obtained, control berries exhibited higher or similar expression than the antioxidant‐mannitol treated berries (Vitalfit) and the alginic acid‐mannitol (SM6) treated berries (only 2022). In 2023 the alginic acid‐mannitol (SM6) treatment slightly enhanced the expression of genes such as *VvPAL*, *VvLDOX, VvUFGT*, and *VvAOMT*.

#### Ripening

**Figure 2:**
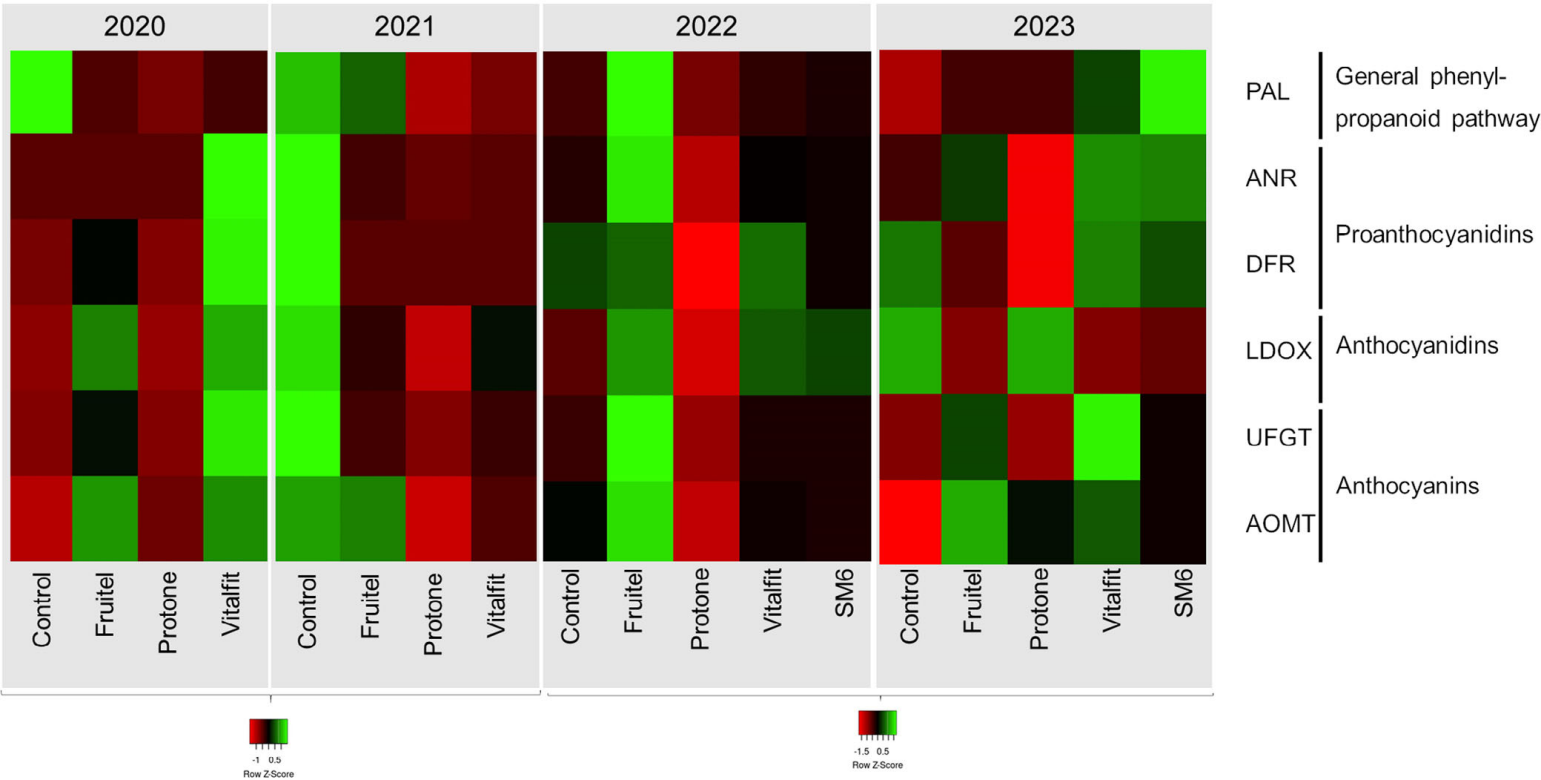
Heatmap of the expression analysis of the genes *VvPAL*, *VvDFR*, *VvANR*, *VvLDOX*, *VvUFGT*, and *VvAOMT* at ripening in the seasons 2020–2024. The treatments analysed were control, ETHEPHON elicitor (Fruitel), ABA elicitor (Protone), antioxidant‐mannitol elicitor (Vitalfit), and alginic acid‐mannitol elicitor (SM6).

At this time‐point, all elicitors had been applied, allowing gene expression analysis across the five treatments (Fig. 2, Supporting Information, Fig. S2). During ripening, various elicitors outperformed control berries significantly, although their effects were not replicable across seasons.

Specifically, the gene *VvPAL*, from the general PP pathway, showed higher expression (significant) in 2023 alginic acid‐mannitol (SM6) treated berries compared to the control grapes.

Two proanthocyanin precursor genes were analysed, *VvDFR* and *VvANR*. The elicitors showed similar trends in both genes, although stronger effects were obtained in *VvDFR*. The antioxidant‐mannitol (Vitalfit) showed higher expression in 2020 (*VvDFR* and *VvANR*) and together with the alginic acid‐mannitol (SM6) elicitor in 2023 (*VvDFR*). In 2022, ethephon elicitor (Fruitel) had the highest expression (significant for *VvDFR*).

Regarding *VvLDOX*, the gene responsible of the anthocyanidin synthesis, effects were very variable across seasons and treatments and no differences were obtained to the control but among treatments (in 2022 between the ethephon elicitor (Fruitel), which was upregulated, and the ABA elicitor (Protone), downregulated).

*VvUFGT* and *VvAOMT* are genes directly involved in the synthesis of anthocyanins, which are coloured stable. They had similar expression trends, although the *VvUFGT* expression was specially affected by the elicitors understudy. In 2022 differences were only obtained among treatments (ethephon elicitor (Fruitel) over ABA elicitor (Protone)). The antioxidant‐mannitol (Vitalfit) showed higher expression than control berries in different seasons (2020, 2023).

In summary, the effect of the elicitors generally started later than veraison. At ripening, some statistical differences were obtained without a repeatable pattern. The antioxidant‐mannitol (Vitalfit) treatment was able to induce gene expression in 2020 and 2023 (in special of *VvDFR* and *VvUFGT*). The ABA elicitor (Protone) did not enhance the expression of polyphenol‐related genes along the experiment.

### Yield parameters

**Figure 3:**
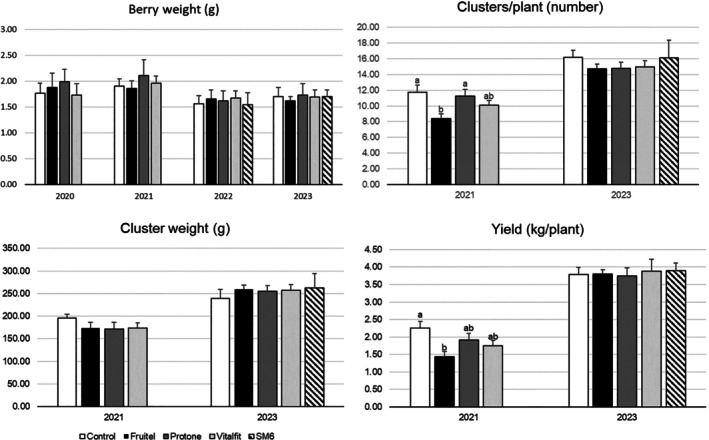
Yield parameters measured as berry weight (in seasons 2020–2023) and clusters per plant, cluster weight, and yield per plant (in seasons 2021 and 2023).

Berry weight and cluster weight were generally not significantly influenced by the elicitor treatments (Fig. 3). However, in the 2021 season, the application of the ethephon elicitor (Fruitel) resulted in a significant reduction in yield that can be attributed to a significant lower number of clusters per plant.

### Grape characterization parameters

**Figure 4:**
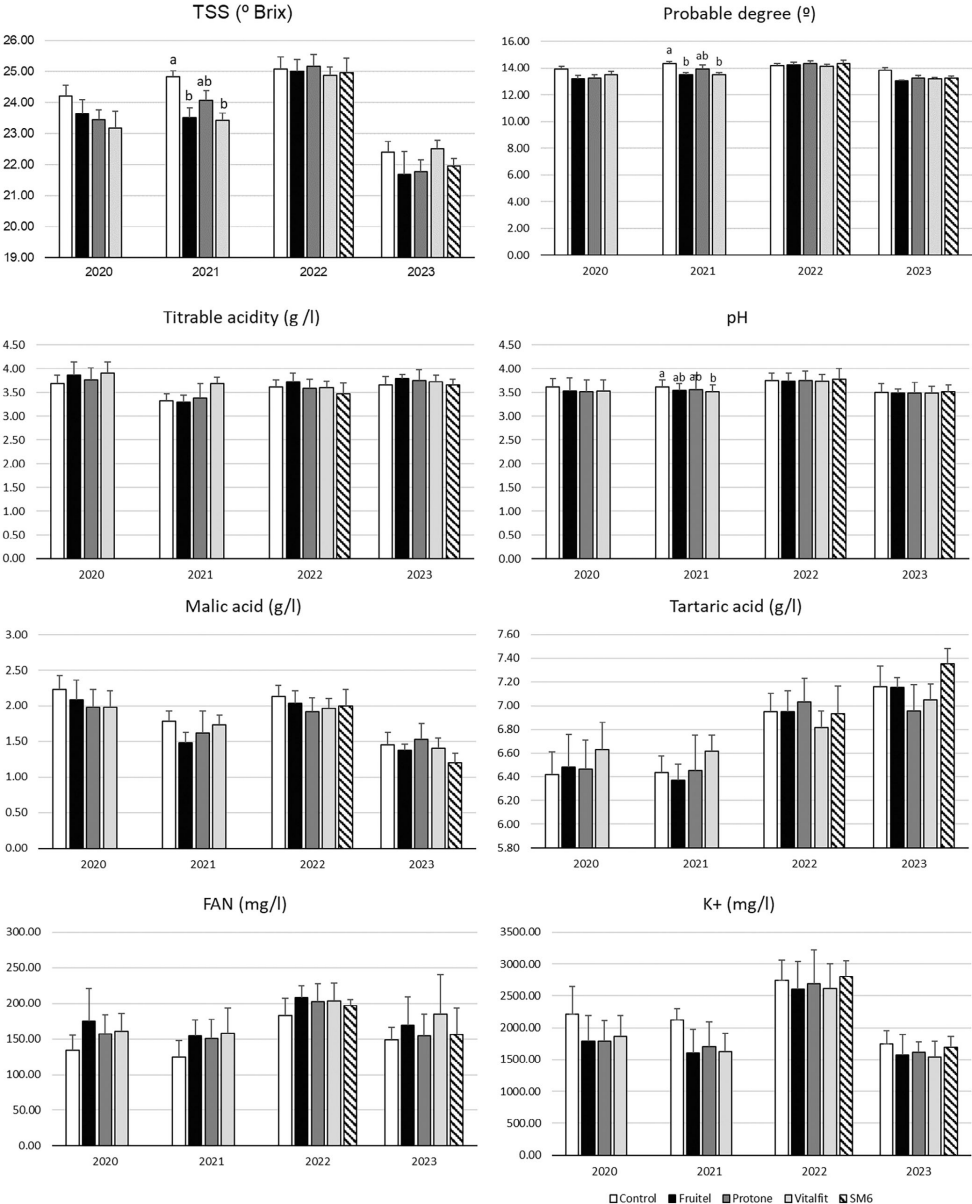
Total soluble solids (TSS), likely alcoholic degree, titratable acidity (TA), pH, malic acid, tartaric acid, FAN, and K+ measured at harvest during the seasons 2020–2024. Data is shown a means ± standard errors (SE). Statistical differences are shown with letters (*P* < 0.05).

None of the parameters measured for grape characterization at harvest (berry weight, likely alcoholic degree, TA, pH, malic acid, tartaric acid, FAN, and K^+^) showed significant differences between treatments consistently over the four seasons (Fig. 4). The treatments did not induce any differences in those parameters during the 2020, 2022, and 2023 at harvest. However, significant seasonal differences were observed in 2021, specifically lower likely alcoholic degree (°)/TSS accumulation (ethephon‐ elicitor (Fruitel) and the antioxidant‐mannitol (Vitalfit)) and lower pH (antioxidant‐mannitol (Vitalfit)).

### Phenolic compound content at harvest

**Figure 5:**
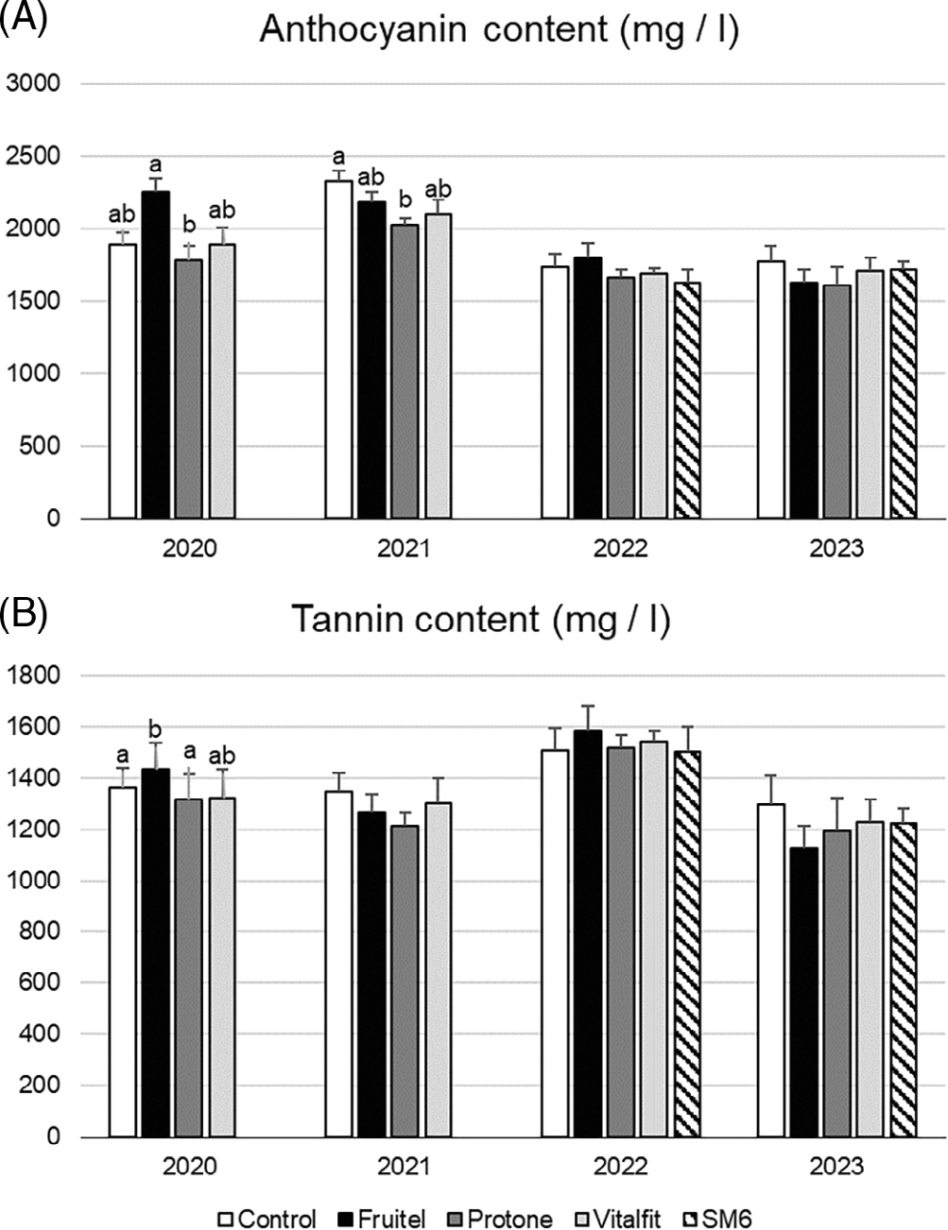
Anthocyanin and tannin content (mg/l) measured at harvest during the seasons 2020–2024. Data is shown a means ± standard errors (SE). Statistical differences are shown with letters (*P* < 0.05).

Regarding tannin and anthocyanin content at harvest, only the ethephon treatment (Fruitel) showed a significant increase in tannin levels compared to the control in 2020 (Fig. 5). In contrast, the ABA treatment resulted in a decrease in anthocyanin accumulation in 2021. No other treatments showed statistically significant differences to the control but ethephon treatment (Fruitel) tended to accumulate more polyphenols than the control in 2022. This highlights the importance of reporting neutral or null results in multi‐year field studies, particularly to help prevent ineffective treatment applications by growers.

Grapes harvested at ripening in the season 2022 were further analysed by UHPLC/MS–MS for anthocyanin, phenolic acid, flavonols, proanthocyanidins, and stilbenes (Supporting Information, Table S2). No statistical differences were obtained between control, antioxidant‐mannitol elicitor (Vitalfit), and alginic acid‐mannitol elicitor (SM6) for any of the polyphenolic compound analysed, mirroring the results obtained by the absorbance method.

In resume, any of the treatments was able to induce a higher accumulation of tannins or anthocyanins across seasons. Only the ethephon treatment (Fruitel) was improving the tannin accumulation in season 2020 (significant) and 2022 (non‐significant).

### Ratio between anthocyanin and TSS

**Figure 6:**
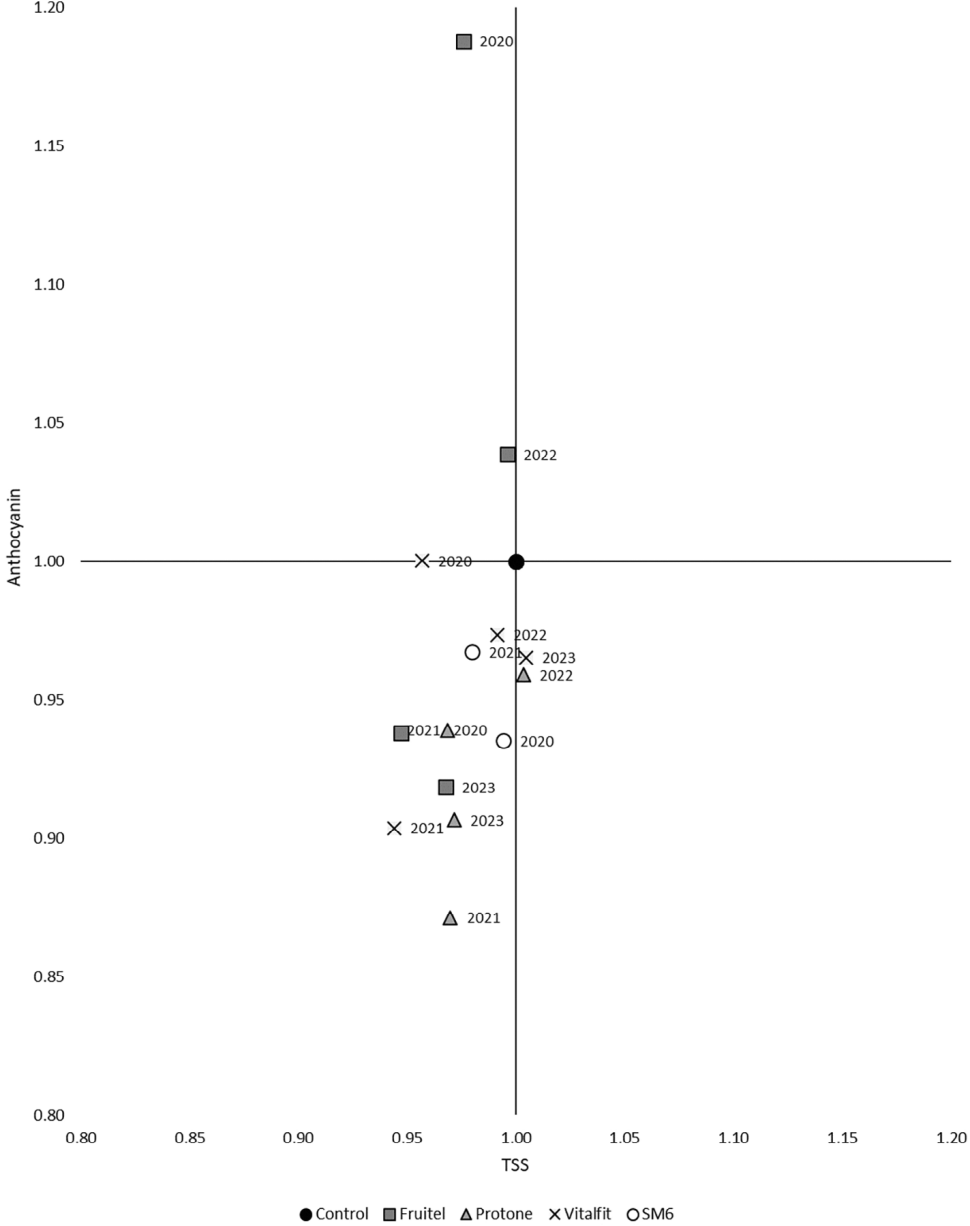
Relation between anthocyanin accumulation and TSS content (°Brix) at harvest. Data shows the ratio standardized with the control: $\frac{\frac{\left(\frac{\mathrm{mg}\;\text{anthocyanin}}{l}\right)\text{treatment}}{\left(\frac{\mathrm{mg}\;\text{anthocyanin}}{l}\right)\text{control}}}{\frac{\left({}^{\circ}\text{brix}\right)\text{treatment}}{\left({}^{\circ}\text{brix}\right)\text{control}}}$. Each point represents one season (2020–2024).

There is a direct relation between sugar and anthocyanin accumulation, as the former triggers the latter through osmotic stress. However, the aim of the application of elicitors here was to decouple the accumulation of these compounds in favour of polyphenols. As seen in the graph (Fig. 6), the treatments contributed to this aim. ethephon‐ elicitor (Fruitel) was the only treatment that increased anthocyanins over TSS (2020 and 2022). In the other two seasons, the treatment resulted in lower sugar and anthocyanin content compared to the control berries. Generally, the other three treatments accumulated less sugars and anthocyanins than the control grape, except of ABA‐treatment (Protone) and antioxidant‐mannitol (Vitalfit) which enhanced sugars in specific seasons.

Overall, the ethephon elicitor (Fruitel) was the only treatment that altered the sugar‐to‐anthocyanin ratio, favouring the latter.

## DISCUSSION

In this study, we evaluated the effect of four different elicitors—two natural and two PH‐based—over four seasons on the accumulation of anthocyanins in the Tempranillo cultivar. Growers and wineries increasingly search for products promoting the accumulation of anthocyanins over sugars. However, it is well‐known that environmental factors and cultivar characteristics can significantly influence these outcomes, elevating the role of field studies to determine the most effective elicitors even if results are often not evident.^32^, ^45^, ^59^

Our results highlight that the use of elicitors is a complex aspect of cultural management, as suggested by other multi‐year field studies.^59^, ^70^, ^93^, ^94^ The modest seasonal increases or lack of significant changes observed with the four treatments tested, do not support recommendations for growers under the same conditions (e.g., variety, climate, doses, and applications). In particular, none of the treatments achieved the primary goal of increasing anthocyanin accumulation. However, the subtle differences observed in our experiment provide valuable insights into the interactions between elicitors and the complex environmental conditions (both plant and field), which could be scientifically leveraged.

### Ethephon application and its effect on TSS accumulation in Tempranillo

Ethephon (Fruitel) was the treatment that induced the most significant responses, including increased tannin content (2020), reduced yield and number of clusters (2021), decreased likely alcoholic degree (2021), enhanced several PP genes expression (2022), and lowered the TSS/anthocyanin ratio (2020 and 2022). Among those changes, likely alcoholic degree reduction is a remarkable outcome. In that season, yield also declined, indicating that the decrease in TSS could not be attributed to a dilution effect from increased production.

The role of ET in nonclimateric fruit ripening, is often associated with the later stages, when secondary metabolites are synthesized.^95^ Nonetheless it is also known to influence sugar accumulation.^96^, ^97^ In the present study, we observed that sugar accumulation in Tempranillo was restrained by ET. Previously, López *et al*.^98^ also reported a reduction in likely alcoholic degree in Tempranillo after treatment with a similar ethephon‐based product and under comparable conditions, consistent with findings from other studies involving ethephon applications in this cultivar.^54^, ^99^ Comparable effects have been observed in other varieties such as Riesling^52^ or Cabernet Sauvignon.^100^ These effects have been attributed either to auxin induction around veraison^97^ or to early chlorophyll degradation in later treatments.^52^ Furthermore, the natural elicitor antioxidant‐mannitol treatment (Vitalfit) reached a similar effect in 2021, what should be further investigated.

Given these findings, ET treatments could serve as a valuable tool for improving the anthocyanin‐to‐sugar ratio under climate change conditions by reducing total soluble solids (TSS). However, this effect appears to be variety‐dependent, as contrasting responses have been reported in other *Vitis vinifera* varieties, including Verdejo,^101^ Muscat Hamburg,^102^ Shine Muscat,^29^ and Crimson Seedless.^28^

### ABA and abiotic stress did not induce or inhibited anthocyanin synthesis

The application of ABA did not produce the expected results in Tempranillo grapes. Contrary to its well‐established role in promoting sugar accumulation and anthocyanin biosynthesis during grape berry ripening,^103^, ^104^, ^105^, ^106^, ^107^ ABA treatment (Protone) tended to reduce anthocyanin levels and suppress the expression of related biosynthetic genes. ABA is naturally produced under abiotic stress conditions, such as drought stress, which can enhance the anthocyanin biosynthesis.^108^ Weather conditions in 2022 were notably stressful, with limited rainfall, particularly in July when treatments were applied. Under such conditions, it is plausible that endogenous ABA levels were already elevated, potentially reducing the efficacy or even counteracting the effects of exogenously applied ABA.

Similarly, treatment with mannitol—known to induce osmotic stress and stimulate ABA biosynthesis in grapevines^76^ —did not produce beneficial effects. In our study, the use of a higher concentration of mannitol with the alginic acid‐mannitol elicitor (SM6), failed to improve or even diminished the outcomes observed with the antioxidant‐mannitol elicitor (Vitalfit), which contained less mannitol. These findings suggest that high concentrations of ABA‐inducing elicitors may negatively affect secondary metabolism in Tempranillo grapes, mirroring the effects seen with direct ABA application.

### Seasonal variability

A major challenge in the use of elicitors is their inconsistent efficacy across growing seasons. In this study, treatment effects varied considerably between years, particularly for the two most promising products: the ethephon‐based treatment (Fruitel) and the antioxidant–mannitol elicitor (Vitalfit). Such variability is a well‐known limitation in multi‐year field trials and is often linked to environmental factors. Although difficult to decipher, in this section we will focus on the most likely factors contributing to this variability.

Meteorological conditions significantly affect elicitor responses.^101^, ^109^ During the study period (2020–2023), notable climatic variability was observed (Table S1). In particular, 2022 experienced extreme summer temperatures exceeding 40 °C. Since ethephon releases ET in a temperature‐dependent manner, excessively rapid ET release under high temperatures can reduce treatment efficacy due to poor plant assimilation.^110^ But on that year the ethephon‐treatment (Fruitel) upregulated some PP genes, contrary to expectations. This could be explained by hormonal crosstalk: thermal stress may have enhanced endogenous ABA levels, potentially acting synergistically with ET.^99^, ^111^ In contrast, the antioxidant‐mannitol product (Vitalfit) was more effective in seasons with milder weather (2020 and 2023), with stronger PP gene expression, what suggests that moderate conditions may better support the oxidative signalling pathways targeted by this treatment.

Grape water content may also contribute to the seasonal variability observed in our data. Although phenolic analyses were based on fresh weight (Cromoenos method), differences in berry water content—driven by climate—can influence the apparent concentration of secondary metabolites.^112^ Hormonal treatments can further affect water content through their impact on stomatal behaviour; ABA, for instance, promotes stomatal closure, potentially leading to higher berry turgor.^113^ Indeed, berries treated with Protone (ABA) tended to have higher weights (not significant) in 2020, 2021, and 2023, which could partly explain the muted phenolic responses observed. Therefore, future studies would benefit from employing a method that allows analysis based on both fresh and dry weight, providing a more accurate assessment of treatment effects.

Moreover, the potential induction of alternative phenolic branches must be considered. For instance, stilbene compounds, such as resveratrol, are stress‐induced phytoalexins commonly associated with biotic stress responses.^114^ However, their synthesis can also be triggered by abiotic stresses—including wounding, UV, drought, and heat—and is hormonally regulated by MeJA, SA, ET, and ABA.^115^, ^116^ Although under mild conditions in 2020 no differences where obtained, future research should explore whether certain elicitors preferentially activate stilbene (i.e., phytoalexin) pathways rather than those linked to pigment biosynthesis.

In conclusion, the hormonal ethephon treatment (Fruitel) produced more pronounced effects but its efficacy was strongly season‐dependent, showing greater results under temperature stress conditions. Moreover, this and other studies suggest that ethephon can reduce total soluble solids (TSS) accumulation in Tempranillo grapes what might be desirable in a climate change scenario. Regarding natural‐based elicitors, they demonstrated enhanced gene expression during specific seasons with typical climatic conditions for the region. Notably, the antioxidant‐mannitol elicitor (Vitalfit) showed positive short‐term results. However, these effects appeared to be transient and did not result in significant differences in anthocyanin accumulation at harvest. Our findings highlight the need for further investigation into the mechanisms of natural‐based elicitors—particularly their impact on other polyphenols such as stilbenes—to improve berry quality under field conditions. They also underscore the potential of these elicitors for field crop optimization, while emphasizing the importance of multi‐year studies to validate their efficacy before making commercial recommendations.

## FUNDING INFORMATION

Open Access funding provided by Universidad Pública de Navarra.

## Supporting information


**Figure S1.** Expression analysis of the genes *VvPAL*, *VvDFR*, *VvANR*, *VvLDOX*, *VvUFGT* and *VvAOMT* at veraison (>60% of grapes coloured) in the seasons 2020–2024. The treatments analysed were control, antioxidant‐mannitol product (Vitalfit), alginic acid‐mannitol product (SM6). Statistical differences are shown with letters (*P* < 0.05).

**Figure S2.** Expression analysis of the genes *VvPAL*, *VvDFR*, *VvANR*, *VvLDOX*, *VvUFGT*, and *VvAOMT* at ripening in the seasons 2020–2024. The treatments analysed were control, antioxidant‐mannitol product (Vitalfit), alginic acid‐manitol product (SM6), Ethephon product (Fruitel), and ABA product (Protone). Statistical differences are shown with letters (*P* < 0.05).

**Table S1.** Weather conditions during the growing seasons from 2019–2020 to 2022–2023. Weather data were obtained from the SIAR weather station in Olite (#11, Navarra, Spain) located close to the research site.

**Table S2.** Anthocyanin, phenolic acid, flavonols, proanthocyanidins, and stilbenes contents analysed by UHPLC/MS–MS in grapes harvested at ripening in the season 2022. ns, non‐significant.

## Data Availability

Data sharing not applicable to this article as no datasets were generated or analysed during the current study.
