# Supplementary figures and images for: Four‐year field study reveals variable effects of phytohormone‐ and natural‐based elicitors on anthocyanin metabolism in Tempranillo grapes
Source: J Sci Food Agric. 2025 Aug 8;105(14):7913–25. doi: 10.1002/jsfa.70050 (PMC12509050; doi:10.1002/jsfa.70050)

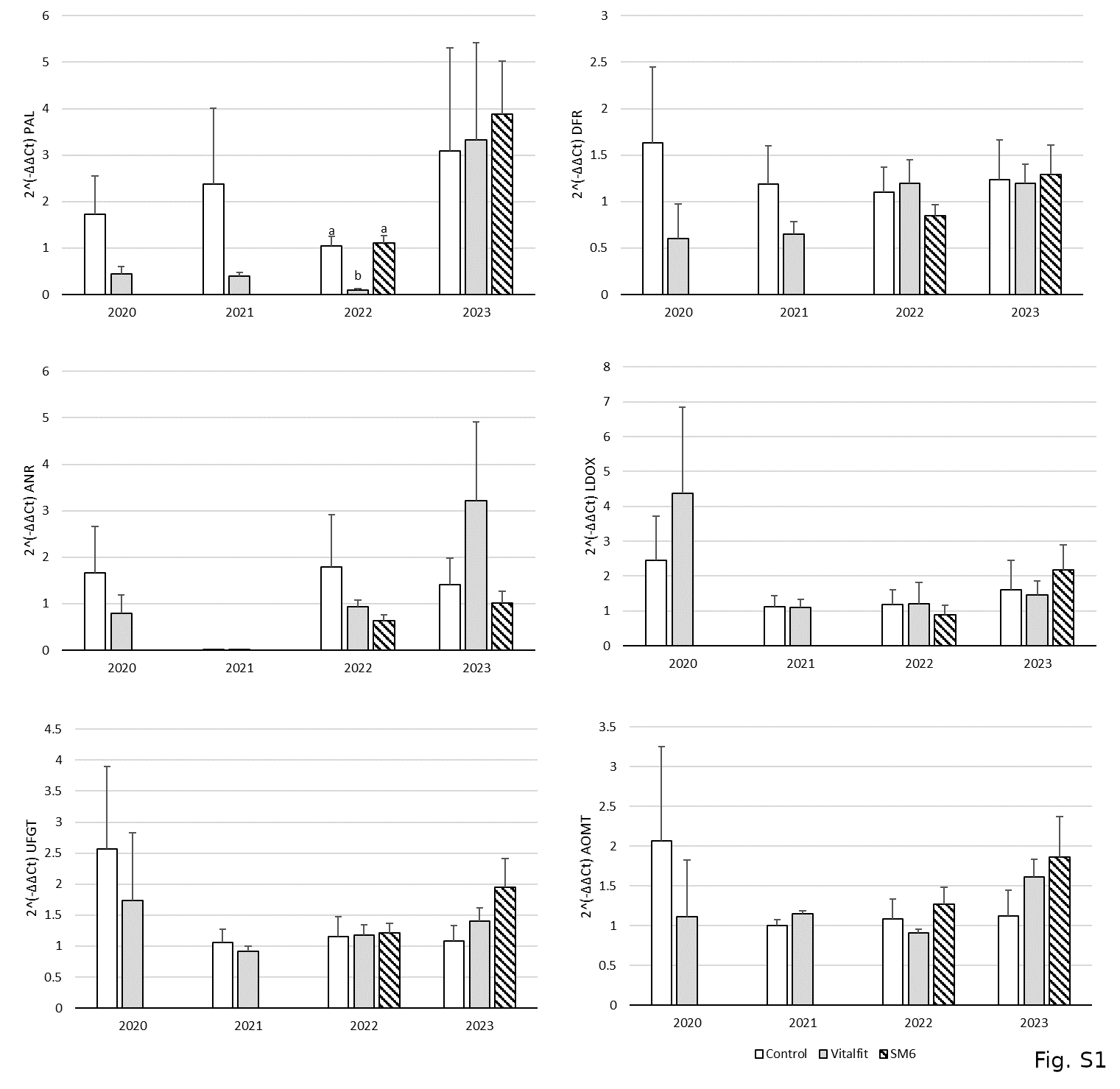

Supplement: Supplementary file 1 — Figure S1. Expression analysis of the genes VvPAL, VvDFR, VvANR, VvLDOX, VvUFGT and VvAOMT at veraison (>60% of grapes coloured) in the seasons 2020–2024. The treatments analysed were control, antioxidant‐mannitol product (Vitalfit), alginic acid‐mannitol product (SM6). Statistical differences are shown with letters (P < 0.05). [file JSFA-105-7913-s002.jpg]

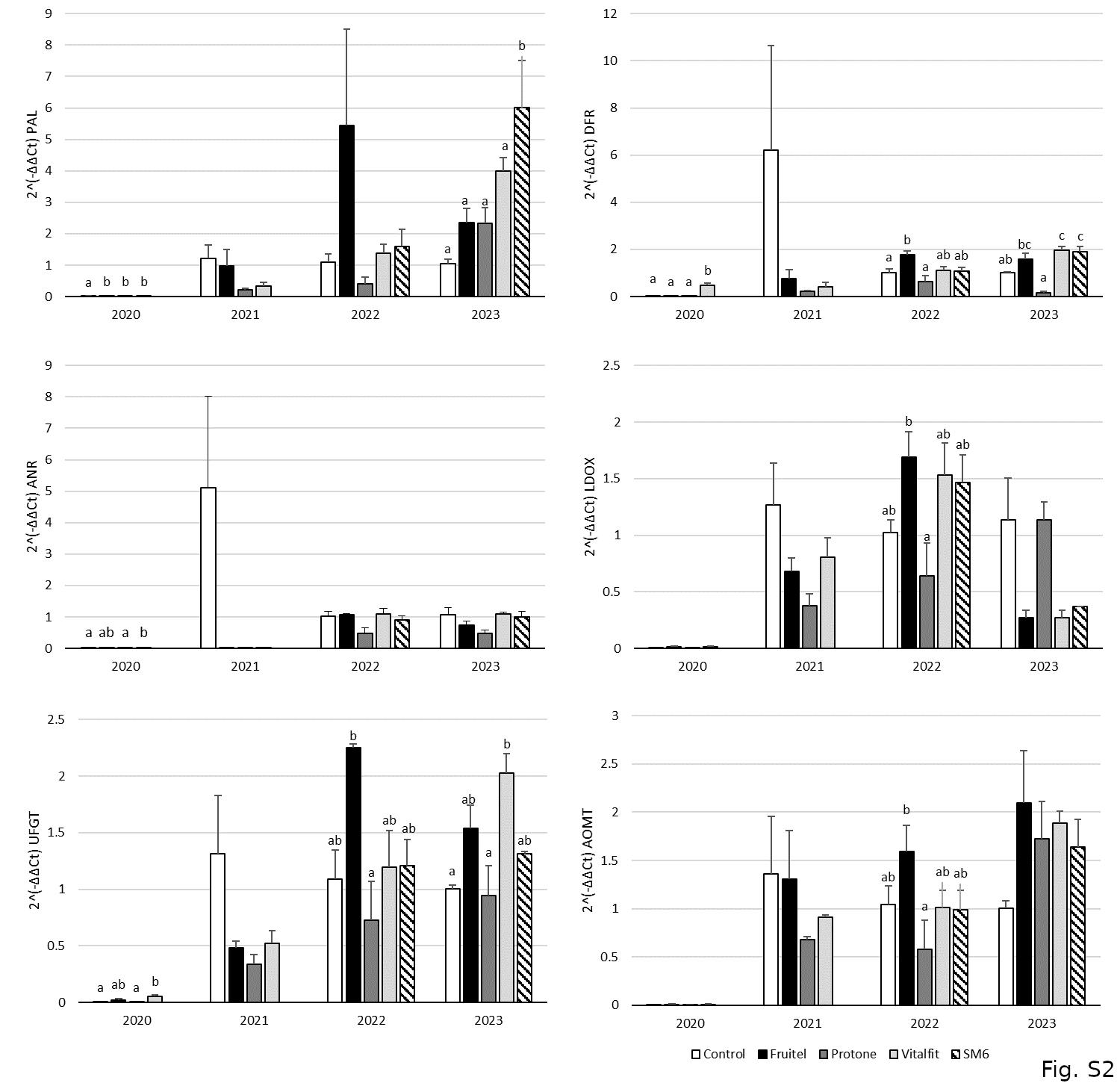

Supplement: Supplementary file 2 — Figure S2. Expression analysis of the genes VvPAL, VvDFR, VvANR, VvLDOX, VvUFGT, and VvAOMT at ripening in the seasons 2020–2024. The treatments analysed were control, antioxidant‐mannitol product (Vitalfit), alginic acid‐manitol product (SM6), Ethephon product (Fruitel), and ABA product (Protone). Statistical differences are shown with letters (P < 0.05). [file JSFA-105-7913-s003.jpg]
