# Supplementary material for: Four‐year field study reveals variable effects of phytohormone‐ and natural‐based elicitors on anthocyanin metabolism in Tempranillo grapes
Source: J Sci Food Agric. 2025 Aug 8;105(14):7913–25. doi: 10.1002/jsfa.70050 (PMC12509050; doi:10.1002/jsfa.70050)
Supplement: Supplementary file 3 — Table S1. Weather conditions during the growing seasons from 2019–2020 to 2022–2023. Weather data were obtained from the SIAR weather station in Olite (#11, Navarra, Spain) located close to the research site. [file JSFA-105-7913-s001.docx]

| **Month** | | | | | | | | | | | | | |
| --- | --- | --- | --- | --- | --- | --- | --- | --- | --- | --- | --- | --- | --- |
|  | October | November | December | January | February | March | April | May | June | July | August | September |  |
| Year | **Mean daily temperature (°C)** | | | | | | | | | | | | Mean |
| 2019-2020 | 15.6 | 8.7 | 7.5 | 5.1 | 9.6 | 9.5 | 13.3 | 18.1 | 18.7 | 22.8 | 22.5 | 19.1 | 14.2 |
| 2020-2021 | 12.7 | 10.0 | 6.2 | 5.3 | 9.7 | 9.6 | 11.1 | 15.3 | 19.9 | 22.1 | 22.1 | 19.2 | 13.6 |
| 2021-2022 | 14.0 | 8.2 | 5.4 | 3.1 | 7.7 | 9.5 | 11.1 | 18.4 | 22.9 | 24.2 | 24.3 | 19.3 | 14.0 |
| 2022-2023 | 18.3 | 10.4 | 7.8 | 4.9 | 5.4 | 11.7 | 14.1 | 15.9 | 21.0 | 22.7 | 23.7 | 20.4 | 14.7 |
| Year | **Minimum daily temperature (°C)** | | | | | | | | | | | | Mean |
| 2019-2020 | 6.1 | -0.5 | -1.5 | -3.4 | -0.1 | 0.0 | 1.5 | 8.0 | 7.2 | 11.1 | 9.0 | 5.6 | 3.6 |
| 2020-2021 | 1.1 | -1.2 | -2.6 | -5.4 | 2.3 | -0.5 | 0.2 | 3.1 | 9.2 | 10.8 | 10.2 | 9.4 | 3.0 |
| 2021-2022 | 1.6 | 0.3 | -1.4 | -5.5 | -3.9 | -1.9 | -2.1 | 7.2 | 10.2 | 10.0 | 13.3 | 7.4 | 2.9 |
| 2022-2023 | 7.1 | 0.3 | -1.0 | -2.4 | -4.2 | -3.5 | 1.2 | 6.4 | 11.8 | 11.7 | 11.2 | 7.0 | 3.8 |
| Year | **Maximum daily temperature (°C)** | | | | | | | | | | | | Mean |
| 2019-2020 | 28.1 | 21.1 | 16.2 | 18.5 | 20.3 | 23.2 | 22.8 | 31.3 | 33.6 | 37.9 | 37.8 | 32.4 | 26.9 |
| 2020-2021 | 24.5 | 22.5 | 13.9 | 18.1 | 18.2 | 22.9 | 23.5 | 30.5 | 34.3 | 38.5 | 40.8 | 32.9 | 26.7 |
| 2021-2022 | 24.6 | 17.5 | 15.5 | 13.8 | 20.8 | 18.0 | 24.8 | 33.3 | 39.1 | 39.7 | 37.8 | 34.3 | 26.6 |
| 2022-2023 | 29.7 | 21.5 | 17.1 | 15.4 | 19.5 | 26.8 | 28.6 | 28.5 | 35.4 | 38.5 | 39.5 | 32.2 | 27.7 |
| Year | **Precipitation (mm)** | | | | | | | | | | | | Total |
| 2019-2020 | 37.2 | 129.2 | 38.0 | 35.4 | 4.0 | 102.0 | 94.8 | 71.8 | 81.4 | 13.4 | 17.9 | 6.4 | 631.5 |
| 2020-2021 | 23.3 | 8.0 | 48.7 | 39.4 | 36.8 | 7.4 | 39.0 | 24.2 | 64.0 | 0.2 | 2.4 | 90.4 | 383.8 |
| 2021-2022 | 25.8 | 93.4 | 53.0 | 25.4 | 2.4 | 34.6 | 52.2 | 14.6 | 14.4 | 80.2 | 8.8 | 25.8 | 430.6 |
| 2022-2023 | 9.0 | 39.0 | 52.2 | 70.6 | 23.0 | 1.0 | 18.2 | 12.4 | 51.4 | 23.0 | 1.0 | 76.4 | 377.2 |
| Year | **Global radiation (MJ/m2)** | | | | | | | | | | | | Total |
| 2019-2020 | 12.3 | 6.3 | 5.8 | 5.9 | 11.5 | 14.2 | 17.1 | 24.2 | 23.8 | 28.0 | 23.4 | 18.4 | 191.0 |
| 2020-2021 | 11.4 | 7.5 | 5.2 | 6.7 | 9.3 | 16.5 | 18.4 | 23.6 | 25.3 | 26.2 | 24.5 | 16.2 | 190.8 |
| 2021-2022 | 13.2 | 8.2 | 4.1 | 9.1 | 12.1 | 11.4 | 19.6 | 26.0 | 26.7 | 28.3 | 23.9 | 18.1 | 200.7 |
| 2022-2023 | 11.4 | 7.1 | 5.1 | 7.5 | 11.7 | 15.7 | 21.4 | 24.0 | 23.6 | 26.2 | 24.5 | 17.4 | 195.5 |

Table S1
