# Supplementary material for: Four‐year field study reveals variable effects of phytohormone‐ and natural‐based elicitors on anthocyanin metabolism in Tempranillo grapes
Source: J Sci Food Agric. 2025 Aug 8;105(14):7913–25. doi: 10.1002/jsfa.70050 (PMC12509050; doi:10.1002/jsfa.70050)
Supplement: Supplementary file 4 — Table S2. Anthocyanin, phenolic acid, flavonols, proanthocyanidins, and stilbenes contents analysed by UHPLC/MS–MS in grapes harvested at ripening in the season 2022. ns, non‐significant. [file JSFA-105-7913-s004.docx]

|  | **Polyphenols (mg/Kg fresh weight)** | | | | | **Anthocyanin families (mg/Kg fresh weight)** | | | | | **Flavonol families (mg/Kg fresh weight)** | | | | | |
| --- | --- | --- | --- | --- | --- | --- | --- | --- | --- | --- | --- | --- | --- | --- | --- | --- |
| **Treatment** | **Total anthocyanins** | **Phenolic acids** | **Flavonols** | **Proanthocyanidins** | **Stilbenes** | **PT** | **DF** | **PN** | **CY** | **MV** | **ISO** | **KA** | **MI** | **QU** | **LA** | **SI** |
| **Control** | 196.62 ± 8.46 | 1.7 ± 0.3 | 52.48 ± 3.7 | 35.64 ± 3.97 | 0.04 ± 0.01 | 0.13 ± 0 | 0.2 ± 0.01 | 0.07 ± 0 | 0.03 ± 0 | 0.57 ± 0.02 | 0.02 ± 0 | 0.04 ± 0.01 | 0.37 ± 0.02 | 0.47 ± 0.02 | 0.05 ± 0 | 0.03 ± 0 |
| **Vitalfit** | 192.23 ± 8.8 | 1.36 ± 0.18 | 54.06 ± 4.04 | 28.86 ± 3.58 | 0.02 ± 0 | 0.13 ± 0 | 0.2 ± 0.01 | 0.07 ± 0 | 0.04 ± 0 | 0.57 ± 0.01 | 0.02 ± 0 | 0.05 ± 0 | 0.34 ± 0.01 | 0.51 ± 0.01 | 0.05 ± 0 | 0.03 ± 0 |
| **SM6** | 179.6 ± 7.54 | 1.31 ± 0.12 | 47.87 ± 3.4 | 26.35 ± 2.52 | 0.04 ± 0.02 | 0.13 ± 0 | 0.2 ± 0.01 | 0.08 ± 0 | 0.04 ± 0 | 0.56 ± 0.02 | 0.02 ± 0 | 0.05 ± 0 | 0.36 ± 0.02 | 0.49 ± 0.02 | 0.05 ± 0 | 0.03 ± 0 |
| **ANOVA** | ns | ns | ns | ns | ns | ns | ns | ns | ns | ns | ns | ns | ns | ns | ns | ns |

Table S2
